# Supplementary material for: Integrative Analysis of Metabolome and Proteome in the Cerebrospinal Fluid of Patients with Multiple System Atrophy
Source: Cells. 2025 Feb 12;14(4):265. doi: 10.3390/cells14040265 (PMC11853536; doi:10.3390/cells14040265)
Supplement: Supplementary file 1 [file cells-14-00265-s001.zip › cells-3427178-supplementary.pdf]

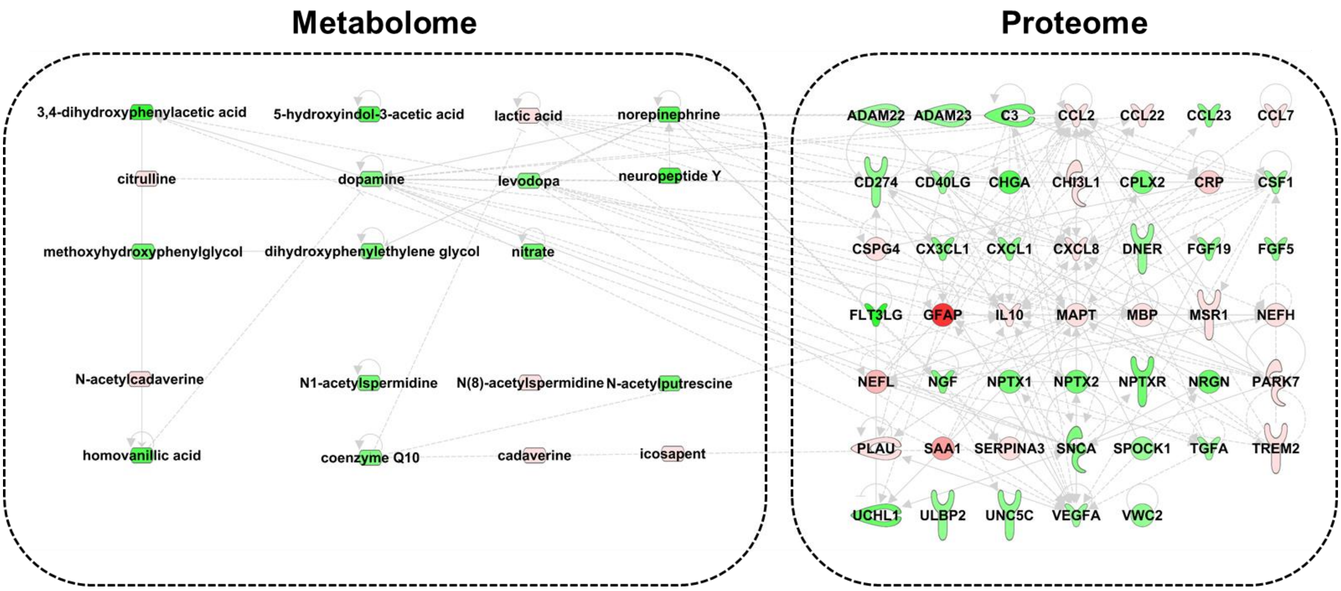

Figure S1. Biological functions and disease-related integrated networks from ingenuity pathway analy sis (IPA). Network of both metabolites and proteins and their associated functions and diseases in the cerebrospinal fluid of patients with MSA.

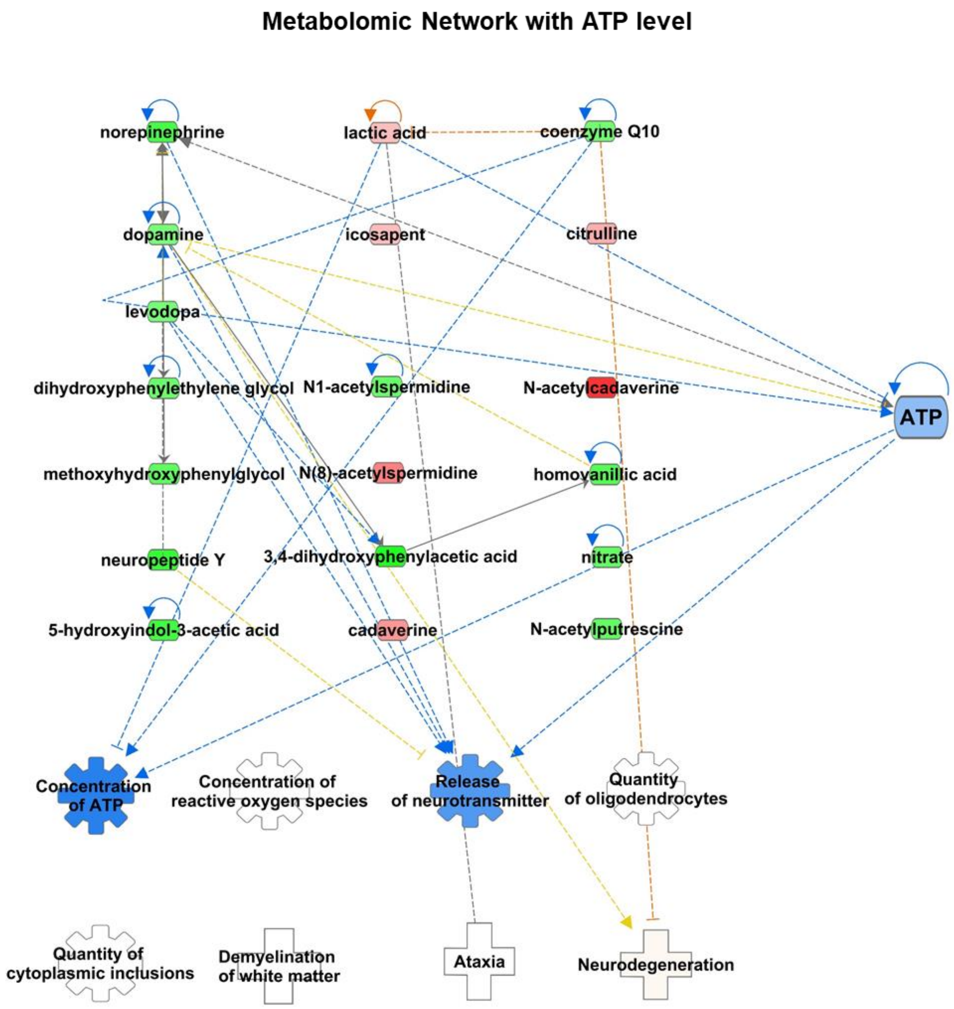

Figure S2. In silico predictions for metabolomic network that shows increased lactic acid directly imp acts ATP levels.

Table S1. Sample size for statistically significantly altered metabolites in CSF of MSA patients

Reference	Sample Size	Metabolite
Goldstein et al, 2012	38 HC, 54 MSA	3,4-dihydroxyphenylalanine<br>Dopamine<br>3.4-dihydroxyphenylglycol<br>3,4-dihydroxyphenylacetic acid
Kuiper et al, 1999	21 HC, 15 MSA	L-Citrulline
Abdo et al, 2007	40 HC, 25 MSA-C,<br>26 HC, 21 MSA-C	Lactate<br>Homovanillic acid
Martignoni et al, 1992	36 HC, 15 MSA	Norepinephrine<br>Neuropeptide Y<br>3-methoxy-4-hydroxyphenyl glycol
Lee et al, 2008	13 HC, 10 MSA	Eicosapentaenoic acid
Compta et al, 2018	15 HC, 20 MSA	Coenzyme Q10
Paik et al, 2010	24 HC, 9 MSA	N^1-Acetylcadaverine<br>N^1- Acetylspermidine<br>N^8- Acetylsperimidine<br>N^1- Acetylputrescine<br>Cadaverine
Nagao et al, 2024	33 HC, 69 MSA	5- Hydroxyindoleacetic acid
Kuiper et al, 1994	20 HC, 14 MSA	Nitrate

Table S2. List of altered metabolites in CSF samples of MSA patients

Metabolite	Expression level	Analysis Method [a]	P value	Reference (PMID Number)
Uric acid	Decrease	Enzyme assay	NS	22998191
Pyruvic acid	Increase	Amino acid analyser	NS	15133823
Nitrite	Decrease	ELISA	NS	8133311
Glutamate	Increase	HPLC	NS	10847559
Glutathione	Increase	HPLC	NS	10526192
Arginine	Increase	HPLC	NS	10847559
Spermidine	Decrease	GC-MS	NS	20515677

[a] Abbreviations: ELISA, enzyme-linked immunosorbent assay; HPLC, high performance liquid chromatography ; GC-MS, gas chromatography with mass spectrometry; NS, not significant.

Table S3. Sample size for proteins altered in CSF of MSA patients

Reference	Sample size	Protein Name
Katzdobler et al, 2024	25 HC, 47 MSA	NFL<br>GFAP
Magdalinou et al, 2014	30 HC, 31 MSA	SYUA<br>CH3L1<br>CCL2
Abdo et al, 2003	36 HC, 10 MSA-C	TAU
Santaella et al, 2020	38 HC, 22 MSA	MBP
Hall et al, 2018	50 HC, 24 MSA	CRP<br>SAA1<br>IL8
Mondello et al, 2022	22 HC, 34 MSA	UCHL1
Herbert et al, 2014	30 HC, 23 MSA	PARK7
Santaella et al, 2020	25 HC, 14 MSA	X3CL1<br>FGF19<br>CD40L<br>PD1L1<br>TGFA<br>CSF1<br>UROK<br>VEGFA<br>CCL23<br>GROA<br>DNER<br>NGF
Compta et al, 2019	15 HC, 39 MSA	CCL7<br>IL10<br>CCL22
Wang et al, 2011	137 HC, 32 MSA	CO3
Shi et al, 2011	137 HC, 32 MSA	FLT3L
Hall et al, 2020	47 HC, 26 MSA	NEUG<br>NFH
Kaiserova et al, 2021	31 HC, 18 MSA	CMGA
Jabbari et al, 2019	34 HC, 38 MSA	FG5<br>MSRE<br>VWC2<br>ADA22<br>UNC5C<br>ADA23<br>T1CN1<br>ULBP2
Schulz et al, 2021	20 HC, 17 MSA	TREM2
Tokutake et al, 2022	20 HC, 50 MSA	CSPG4
Nilsson et al, 2023	48 HC, 31 MSA	NPTX1

		NPTX2<br>NPTXR<br>CPLX2
Furiya et al, 2005	11 HC, 21 MSA	AACT

Table S4. List of altered protein level without statistical significance in CSF of MSA patients

Protein [a]	Accession Number [b]	Expression level	P value	Analysis Method [c]	Reference (PMID Number)
S-100B	P04271	Increase	NS	Immunoluminometric assay	32576494
NSE	P09104	Increase	NS	Immunoluminometric assay	32576494
IL-6	P05231	Increase	NS	Biomarkers kit	30185816
EGF	P01133	Decrease	NS	Multiplex bead assay	31178335
FGF-2	P09038	Increase	NS	Multiplex bead assay	31178335
CCL11	P51671	Increase	NS	Multiplex bead assay	31178335
G-CSF	P09919	Increase	NS	Multiplex bead assay	31178335
GM-CSF	P04141	Decrease	NS	Multiplex bead assay	31178335
IFN-α2	P01563	Increase	NS	Multiplex bead assay	31178335
IFN-γ	P01579	Same	NS	Multiplex bead assay	31178335
IL-13	P35225	Increase	NS	Multiplex bead assay	31178335
IL-15	P40933	Decrease	NS	Multiplex bead assay	31178335
IL-17	Q16552	Same	NS	Multiplex bead assay	31178335
IL-1ra	P18510	Decrease	NS	Multiplex bead assay	31178335
IL-1a	P01583	Same	NS	Multiplex bead assay	31178335
IL-9	P15248	Decrease	NS	Multiplex bead assay	31178335
IL-1b	P01584	Increase	NS	Multiplex bead assay	31178335
IL-2	P60568	Decrease	NS	Multiplex bead assay	31178335
IL-4	P05112	Same	NS	Multiplex bead assay	31178335
IL-5	P05113	Increase	NS	Multiplex bead assay	31178335
IL-7	P13232	Decrease	NS	Multiplex bead assay	31178335
IP-10	P02778	Increase	NS	Multiplex bead assay	31178335
MIP-1α	P10147	Increase	NS	Multiplex bead assay	31178335
MIP-1β	P13236	Increase	NS	Multiplex bead assay	31178335
TNF-α	P01375	Increase	NS	Multiplex bead assay	31178335
TNF-β	P01374	Same	NS	Multiplex bead assay	31178335
CFAH	P08603	Increase	NS	Bead Based Luminex assay	21435440
CCL28	Q9NRJ3	Increase	NS	Proximity Emission Assay	31952511
MCP-2	P80075	Decrease	NS	Proximity Emission Assay	31952511
TGF-β1	P01137	Decrease	NS	ELISA	27756506
SCRN1	Q12765	Increase	NS	MS/MS	36946611
IGF-II	P01344	Increase	NS	SRM assay with MS	34848724
FAT2	Q9NYQ8	Increase	NS	SRM assay with MS	34848724
PMGT1	Q8WZA1	Decrease	NS	SRM assay with MS	34848724
PEDF	P36955	Increase	NS	SRM assay with MS	34848724
CD90	P04216	Decrease	NS	SRM assay with MS	34848724
MEGF8	Q7Z7M0	Decrease	NS	SRM assay with MS	34848724
ECM1	Q16610	Decrease	NS	SRM assay with MS	34848724
NBL1	P41271	Increase	NS	SRM assay with MS	34848724
NRCAM	Q92823	Decrease	NS	SRM assay with MS	34848724
CADH2	P19022	Decrease	NS	SRM assay with MS	34848724
NOE1	Q99784	Increase	NS	SRM assay with MS	34848724

SE6L2	Q6UXD5	Increase	NS	SRM assay with MS	34848724
PLTP	P55058	Increase	NS	SRM assay with MS	34848724
NFASC	O94856	Increase	NS	SRM assay with MS	34848724
SCG2	P13521	Decrease	NS	SRM assay with MS	34848724
ITI-HC5	Q86UX2	Increase	NS	SRM assay with MS	34848724
CD44	P16070	Increase	NS	SRM assay with MS	34848724
SLIK1	Q96PX8	Decrease	NS	SRM assay with MS	34848724
LIGO1	Q96FE5	Decrease	NS	SRM assay with MS	34848724
MMP-2	P08253	Decrease	NS	SRM assay with MS	34848724
PCI	P05154	Decrease	NS	SRM assay with MS	34848724
CPE	P16870	Increase	NS	SRM assay with MS	34848724
CLN5	O75503	Increase	NS	SRM assay with MS	34848724
KPYM	P14618	Increase	NS	SRM assay with MS	34848724
FBLN1	P23142	Increase	NS	SRM assay with MS	34848724
PROS	P07225	Increase	NS	SRM assay with MS	34848724
CAB45	Q9BRK5	Increase	NS	SRM assay with MS	34848724
PCDGM	Q9Y5F6	Decrease	NS	SRM assay with MS	34848724
HMIC	Q9NR34	Increase	NS	SRM assay with MS	34848724
PGRP-L	Q96PD5	Increase	NS	SRM assay with MS	34848724
ATIII	P01008	Increase	NS	SRM assay with MS	34848724
CNTN1	Q12860	Decrease	NS	SRM assay with MS	34848724

[a] The protein symbol indicates the human protein assigned by UniProtKB. [b] The accession number indicates the unique identifier of the protein in UniProtKB. [c] Abbreviations: MS/MS, tandem mass spectrometry; ELISA, enzyme-linked immunosorbent assay; SRM with MS, selected reaction monitoring with mass spectrometry; NS, not significant.

Table S5. List of the top ten inhibited disease and biological functions identified through integrative omics analysis

Disease and Biological Function	Z score	Molecules
Apoptosis of neuroglia	-1.037	CCL2, CX3CL1, NGF, SNCA, TREM2, VEGFA
Apoptosis of neurons	-1.064	APOE, CCL2, CD274, Dopamine, IL10, MAPT, NEFL, NGF, NPTX1, PARK7, SERPINA3, SNCA, TAC1, VEGFA
Neurogenesis of brain	-1.067	CX3CL1, MAPT, SNCA, VEGFA, VWC
Depolarization of neurons	-1.165	Dopamine, NGF, Norepinephrine, TAC1
Proliferation of neuroglia	-1.206	CSF1, CX3CL1, CXCL1, DNER, GFAP, NEFL, NGF, SNCA, TREM2, VEGFA
Proliferation of neuronal cells	-1.404	APOE, CCL2, CSF1, CX3CL1, DNER, FGF5, GFAP, Lactic acid, Levodopa, MAPT, NEFH, NGF, NPTX1, TGFA, VEGFA
Neuronal cell death	-1.452	APOE, CCL2, CD274, CSF1, CX3CL1, CXCL1, CXCL8, Dopamine, FGF5, GFAP, IL10, Lactic acid, Levodopa, MAPT, MBP, NEFH, NEFL, NGF, Norepinephrine, NPTX1, PARK7, SERPINA3, SNCA, TAC1, TGFA, UCHL1, VEGFA
Release of neurotransmitter	-1.692	C3, CPLX2, Dopamine, Levodopa, NGF, Norepinephrine, PARK7, SNCA, TAC1
Density of neurons	-1.777	APOE, CSPG4, Levodopa, MAPT, NEFH, NGF, SNCA, TREM2, UCHL1, VEGFA
Development of neurons	-2.106	ADAM22, APOE, CSF1, CSPG4, CX3CL1, DNER, Dopamine, FGF5, GFAP, Levodopa, MAPT, MBP, NEFH, NEFL, NGF, NPTXR, PLAU, SNCA, SPOCK1, TREM2, UCHL1, UNC5C, VEGFA

Table S6. List of the top ten activated disease and biological functions identified through integrative omics anal ysis

Disease and Biological Function	Z score	Molecules
Degeneration of neurons	0.7	APOE, C3, CCL2, CHGA, CHI3L1, Dopamine, MAPT, NEFH, NGF, PARK7, SNCA, TGFA, UCHL1, UNC5C, VEGFA
Neurotransmission	0.841	APOE, CCL2, CPLX2, CXCL8, Dopamine, Lactic acid, Levodopa, MAPT, MBP, NGF, Norepinephrine, NPTX1, NRGN, PARK7, PLAU, SNCA, UCHL1
Atrophy of neurons	0.882	CCL2, MAPT, NEFH, NGF
Neurodegeneration of axons	0.927	APOE, MAPT, NEFH, NGF, SNCA, VEGFA
Excitation of neurons	0.943	CCL22, Dopamine, Lactic acid, NGF, Norepinephrine, NPTX1, SNCA, UCHL1
Progressive neurological disorder	0.963	5-hydroxyindol-3-acetic acid, ADAM23, APOE, C3, CCL2, CD274, CD40LG, CHGA, CHI3L1, CPLX2, CRP, CSF1, CST3, CXCL1, CXCL8, Dopamine, GFAP, Homovanillic acid, IL10, Lactic acid, Levodopa, MAPT, MBP, NEFH, NEFL, NGF, Norepinephrine, NPTX1, NPTX2, NRGN, PARK7, PLAU, SERPINA3, SNCA, TAC1, TREM2, UCHL1, VEGFA
Survival of cephalic neurons	0.988	APOE, Dopamine, Levodopa, MAPT, NGF, SNCA
Migration of neuroglia	1.708	CCL2, CHI3L1, CSPG4, CX3CL1, CXCL1, GFAP, MAPT, Norepinephrine, TREM2, VEGFA
Firing of neurons	1.715	CSF1, Dopamine, Lactic acid, Levodopa, MAPT, NGF, Norepinephrine
Familial neurological disorder	1.982	5-hydroxyindol-3-acetic acid, ADAM22, APOE, C3, CCL2, CCL7, CHI3L1, Citrulline, CPLX2, CRP, CSPG4 , CST3, CX3CL1, Dopamine, GFAP, Homovanillic acid, IL10, Lactic acid, Levodopa, MAPT, MBP, NEFH, NEF L, NGF, NPTX1, NRGN, PARK7, PLAU, SERPINA3, SNCA, SPOCK1, TAC1, TREM2, UCHL1
